# Author Correction: Perirhinal cortex learns a predictive map of the task environment

**DOI:** 10.1038/s41467-024-50461-3

**Published:** 2024-07-17

**Authors:** David G. Lee, Caroline A. McLachlan, Ramon Nogueira, Osung Kwon, Alanna E. Carey, Garrett House, Gavin D. Lagani, Danielle LaMay, Stefano Fusi, Jerry L. Chen

**Affiliations:** 1 Department of Biomedical Engineering, Boston University, Boston, MA 02215 USA; 2 Center for Neurophotonics, Boston University, Boston, MA 02215 USA; 3 Department of Biology, Boston University, Boston, MA 02215 USA; 4 Center for Theoretical Neuroscience, Columbia University, New York, NY 10027 USA; 5 Department of Neuroscience, Columbia University, New York, NY 10027 USA; 6 Center for Systems Neuroscience, Boston University, Boston, MA 02215 USA

**Keywords:** Cortex, Sensory processing

Correction to: *Nature Communications* 10.1038/s41467-024-47365-7, published online 02 July 2024

In the Acknowledgements section, the grant information ‘the Swartz Foundation (S.F.) should have read ‘the Swartz Foundation (S.F. and R.N.)’. The original article has been corrected.

