# Supplementary material for: Perirhinal cortex learns a predictive map of the task environment
Source: Nat Commun. 2024 Jul 2;15:5544. doi: 10.1038/s41467-024-47365-7 (PMC11219840; doi:10.1038/s41467-024-47365-7)
Supplement: Supplementary file 1 — Supplementary Information [file 41467_2024_47365_MOESM1_ESM.pdf]

Supplementary Information for Perirhinal Cortex Learns a Predictive Map of the Task Environment

David G. Lee[1,2¥], Caroline A. McLachlan[2,3¥], Ramon Nogueira[4,5], Osung Kwon[2,3], Alanna E. Carey[2,3], Garrett House[3], Gavin D. Lagani[3], Danielle LaMay[3], Stefano Fusi[4,5], Jerry L. Chen[1,2,3,6]

[1]*Department of Biomedical Engineering, Boston University, Boston MA 02215, USA*

[2]*Center for Neurophotonics, Boston University, Boston MA 02215, USA.*

[3]*Department of Biology, Boston University, Boston MA 02215, USA*

[4]*Center for Theoretical Neuroscience, Columbia University, New York, NY 10027, USA.*

[5]*Department of Neuroscience, Columbia University, New York NY 10027, USA.*

[6]*Center for Systems Neuroscience, Boston University, Boston MA 02215, USA.*

[¥]These authors contributed equally

[*]*Correspondence*. E-mail: jerry@chen-lab.org (J.L.C.)

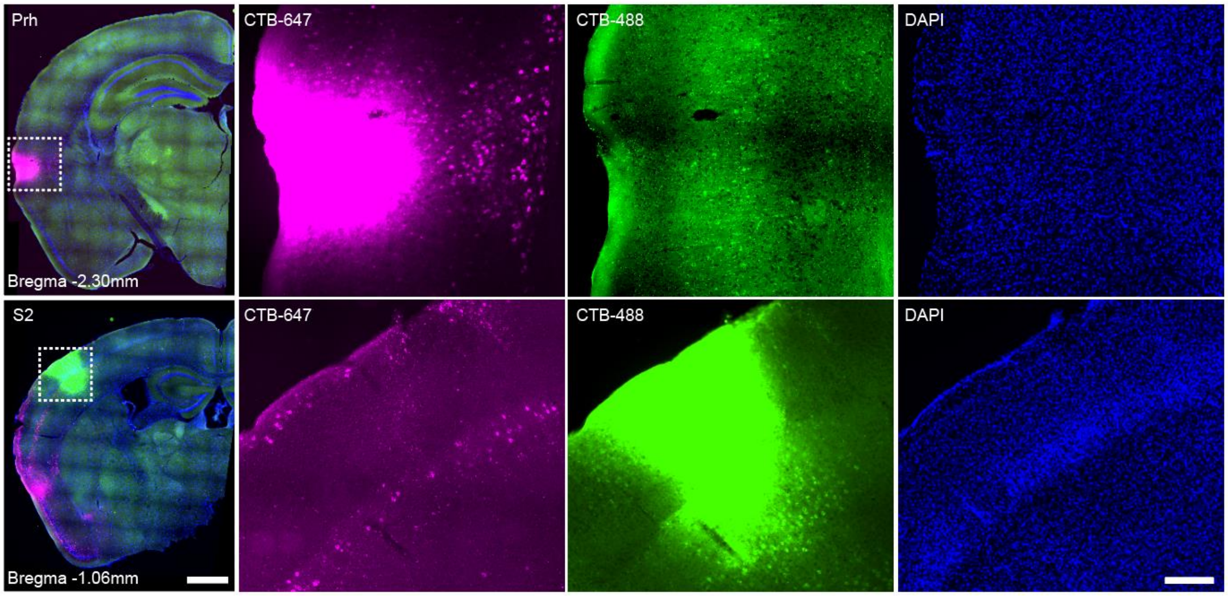

Supplementary Fig. 1. Reciprocal connections between perirhinal cortex and secondary somatosensory cortex. Fluorescent micrographs of coronal sections showing retrograde labeling of projection neurons between perirhinal cortex (CTB-647) and secondary somatosensory cortex (CTB-488). Right panels show magnified view of indicated area in left panel (dotted rectangle). Scale bars: 1mm (left panels), 0.2mm (right panels).

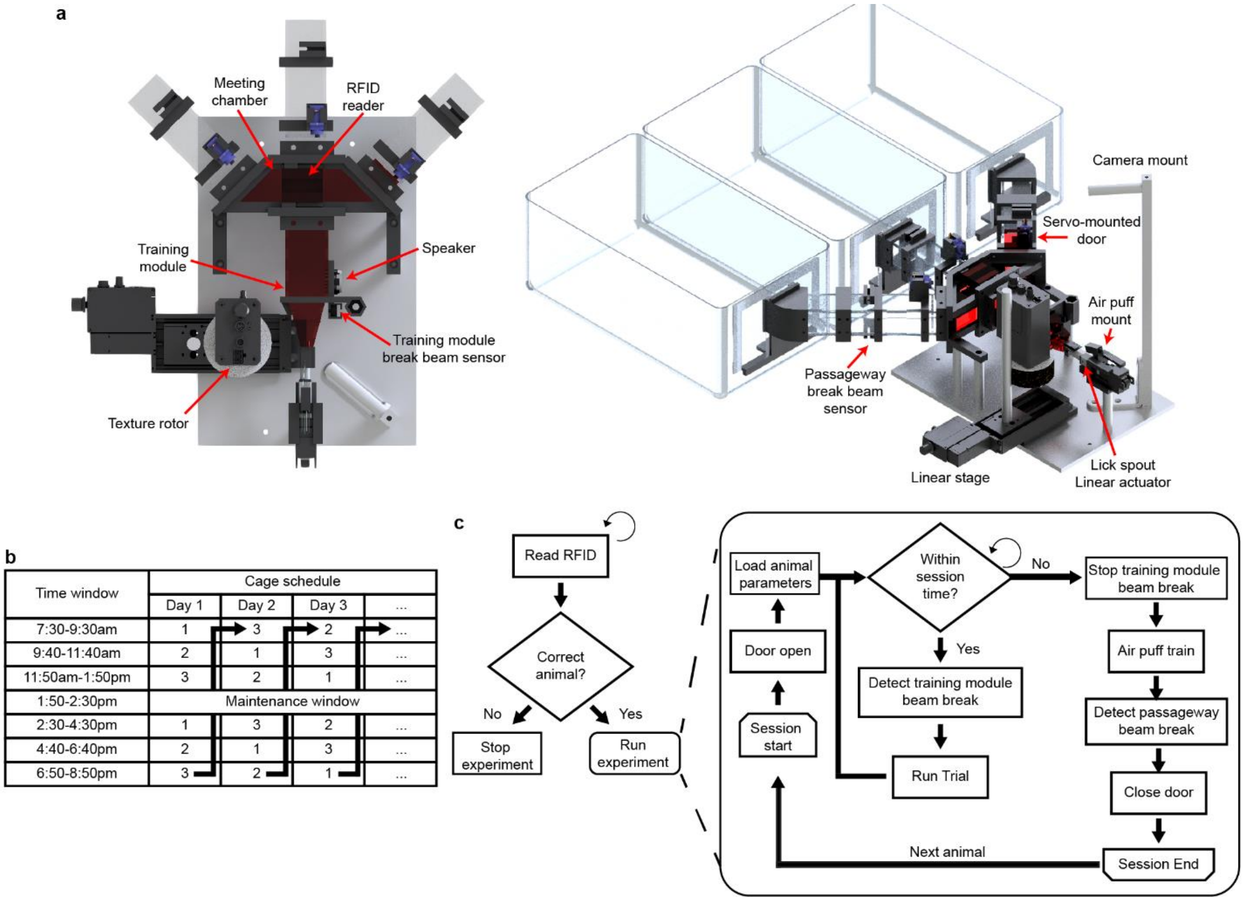

Supplementary Fig. 2. Automated home cage training system. a, Mechanical design of home-cage training system designed to support automated training of three individually housed mice. **b,** Rotating daily timetable used for training three animals (1, 2, 3). **c,** Flow chart for managing individual animals in the training system.

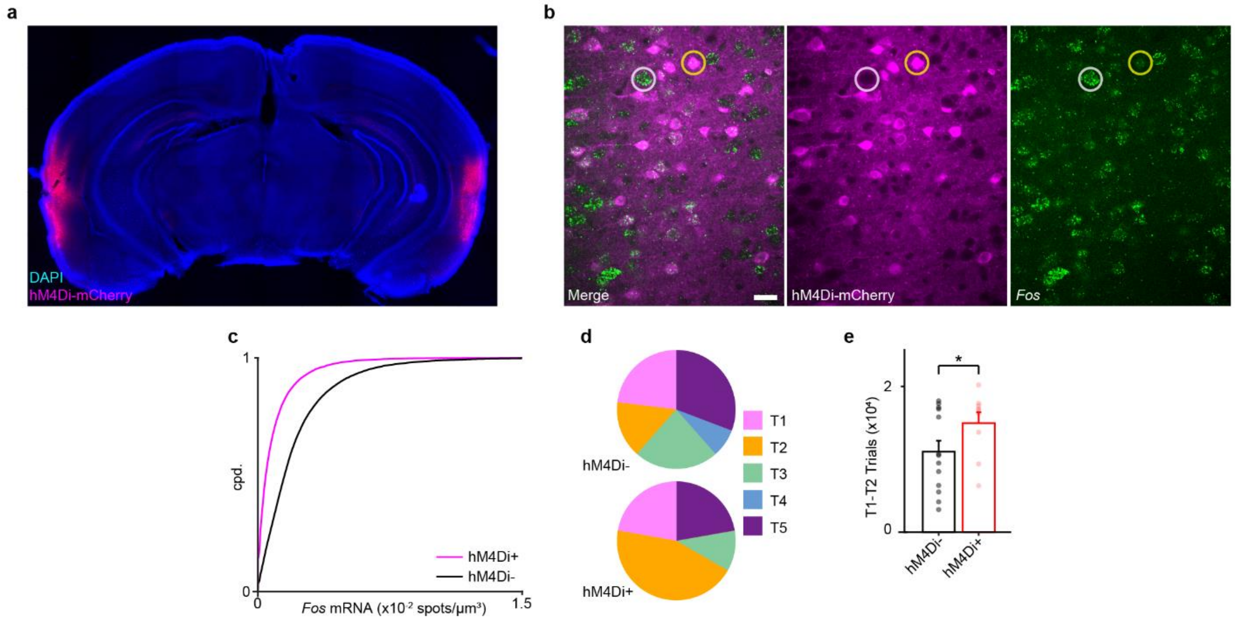

Supplementary Fig. 3. Chemogenetic inactivation of perirhinal cortex. a, Coronal section stained with DAPI (blue) showing bilateral expression of hM4Di-mCherry (magenta) from chemogenetic inactivated animals during home cage task training, representing *n* = 22 animals. **b,** Validation of chronic inactivation of perirhinal cortex by *Fos* mRNA expression. Animals received Compound 21 in drinking water for up to 6 weeks. *Fos* mRNA was visualized using HCR-FISH. Examples of hM4Di-mCherry+ neurons (yellow) with low *Fos* expression versus hM4Di-mCherry- neurons (grey) with high *Fos* expression are shown. **c,** Cumulative distribution of *Fos* expression measured by HCR-FISH in hM4Di-mCherry+ vs. hM4Di-mCherry- neurons. 74.9±3.0% of neurons were hM4Di-mCherry+, *n* = 4 animals. Scale bar: 20 µm. **d,** Distribution of final training stage reached for each animal after 84 training sessions for hM4Di- (top) versus hM4Di+ (bottom) groups. The majority of hM4Di+ animals failed to advance past T2. **e,** Number of trials performed in stages T1-T2 by hM4Di- versus hM4Di+ groups. hM4Di+ animals spent more training time in T1-T2. ($^*P$<0.05, Student's t-test, *n* = 13 hM4Di- animals, 9 hM4Di+ animals). Scale bar = 0.5mm. Error bars = SEM.

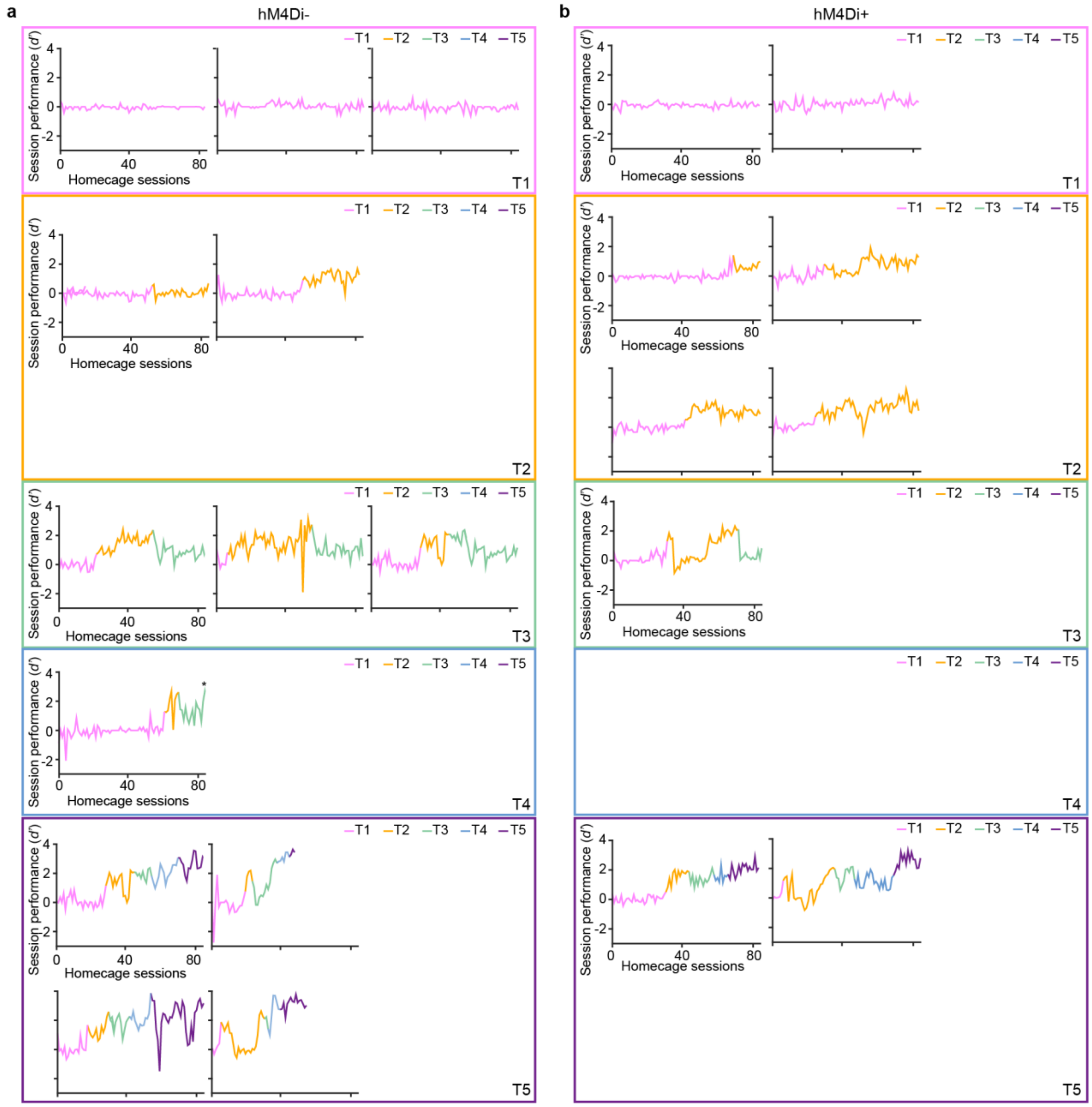

Supplementary Fig. 4. Performance curves for individuals in home cage training task. a, Session performance across training for hM4Di- animals sorted by final training stage reached after 84 sessions. Training was stopped prior to 84 sessions for some animals that reached T5. The majority of hM4Di- animals passed T2. The noted animal (*) reached T4 at session 84. **b,** Session performance across training for hM4Di+ animals sorted by final training stage reached after 84 sessions. The majority of hM4Di+ animals failed to passed T2.

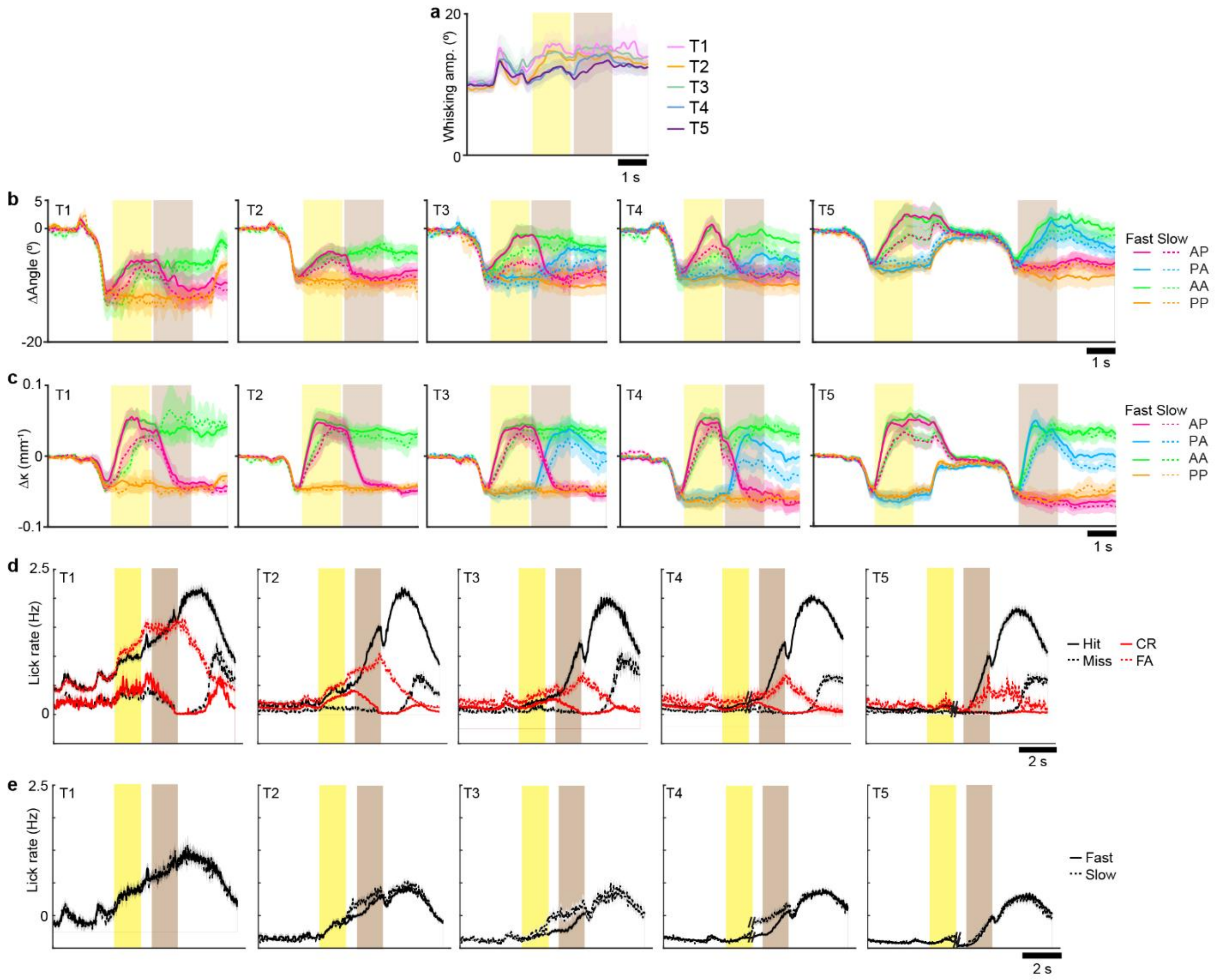

Supplementary Fig. 5. Sensory and motor variables throughout learning. a, Mean whisking amplitude over the trial period averaged across training stages. **b-c,** Mean change in whisker angle [b] and curvature [c] by sorted stimulus condition across training stages. **d-e,** Mean lick rate through the trial period across training stages sorted by choice [d] or stimulus speed [e]. Shaded regions = SEM.

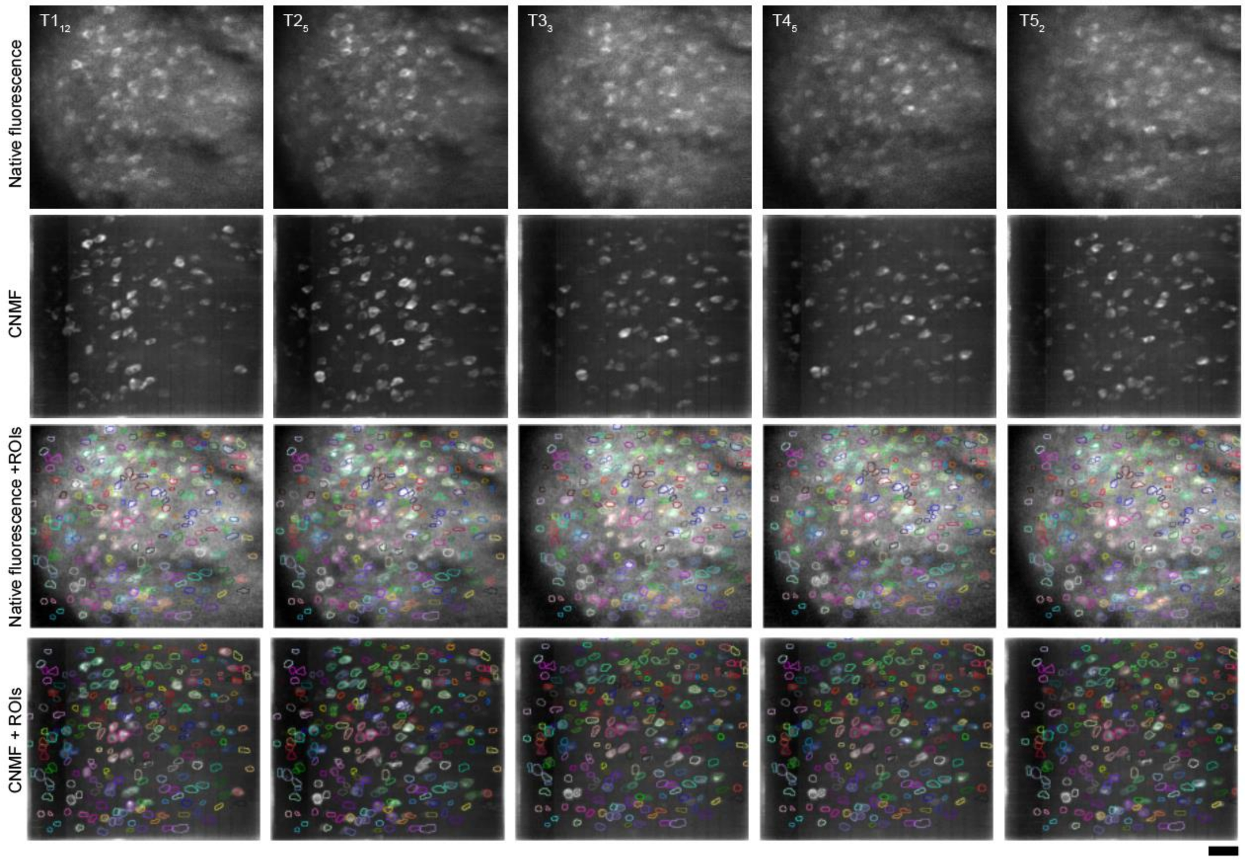

Supplementary Fig. 6. ROI selection across imaging sessions. Examples of ROIs identified throughout the time course of training representing *n*=7 animals imaged for 26-68 consecutive sessions, depending on performance. ROIs were manually identified and segmented by comparing structural images of native RCaMP1.07 fluorescence and images of ‘active’ neurons through constrained non-negative matrix factorization (CNMF) of the image timeseries across the training session. Structural images were used to identify all neurons (active and inactive) in the session while the CNMF images helped to define boundaries of ROIs. Scale bar: 50µm.

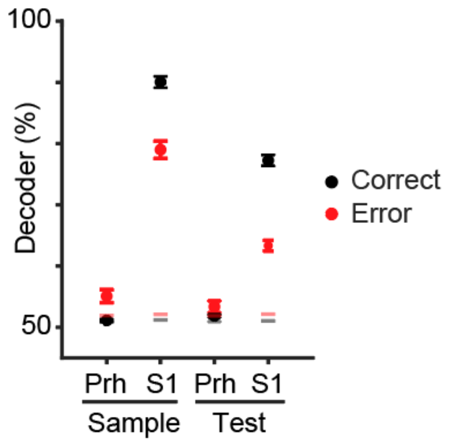

Supplementary Fig. 7. Population encoding of stimulus direction Prh versus S1 in expert animals. Decoder performance on stimulus direction during Sample or Test periods using activity T5 sessions from Prh or S1. S1 neural data was obtained from previous study[1]. Separate decoders were trained and tested using Correct (Hit and Correct Rejection) or Error (False Alarm and Miss) trials. Error bars = SEM. Red and gray bars = 95th percentile of shuffled distribution on Error and Correct trials, respectively.

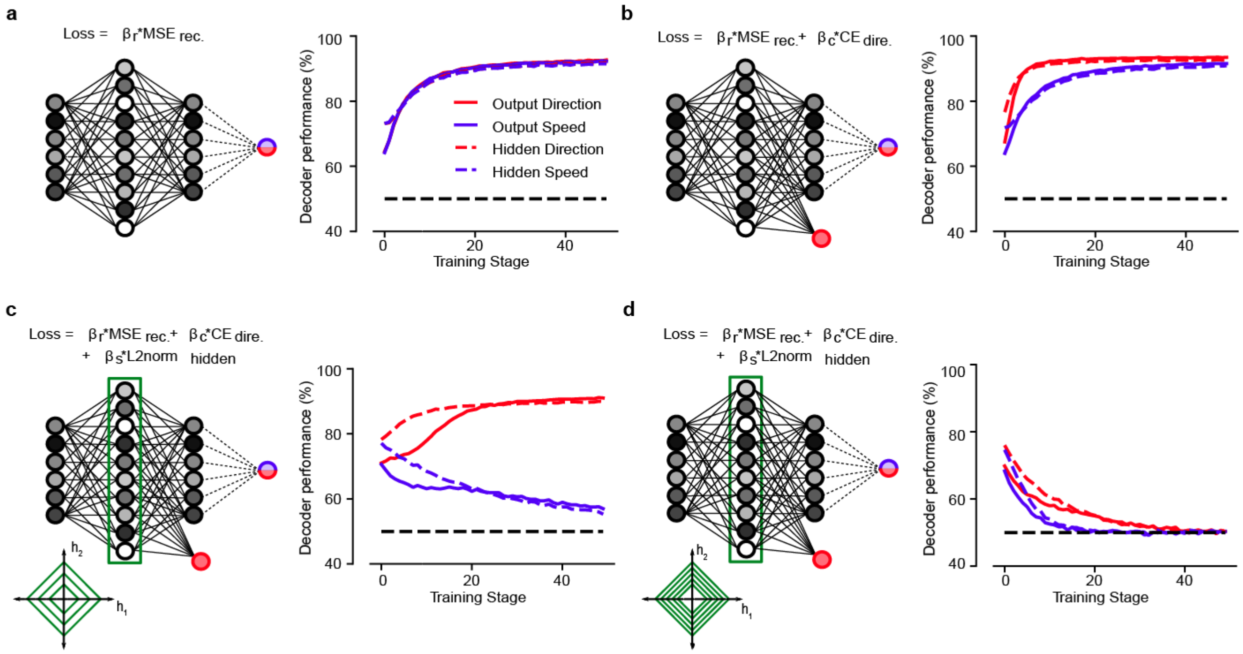

Supplementary Fig. 8. Alternate models produce different decoding performance of direction and speed across learning. Decoding performance for direction of motion (red) and speed (blue) of a linear classifier that reads out the output (solid line) and hidden (dashed line) layers of the autoencoder (logistic regression, sci-kit learn). **a,** Results from an autoencoder trained to minimize only reconstruction loss (Mean Squared Error, MSE). Direction and speed show very similar dynamics throughout learning. **b,** Model with an extra term added in the loss function (cross-entropy loss, CE) to minimize the classification error on direction of motion. The decoding performance of the classifier is higher for the direction of motion. **c,** Model with an additional term on the loss function to limit the activity of the hidden layer in the autoencoder (L1-norm). This configuration of network parameters is similar to **Fig. 3b** with the linear classifier reading out from the difference between the reconstructed output and the input. The model discards information about speed and only keeps information about direction of motion. **d,** Same network configuration as [c] with a sparsity penalty that is too large. The network discards information about both speed and direction of motion. Error bars in all panels correspond to SEM across independent simulations (n = 100).

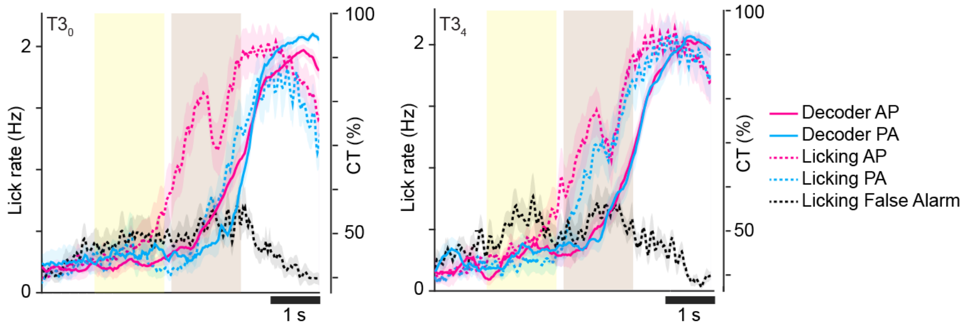

Supplementary Fig. 9. Licking behavior during T3 sessions does not align with population-level stimulus-reward associations. Cross-temporal decoder performance trained on report activity for the rewarded AP or PA condition overlaid with mean licking rate for AP, PA, and false alarm conditions during the $T3_0$ or $T3_4$ session. Decoder performance lags licking behavior. Shaded region = SEM.

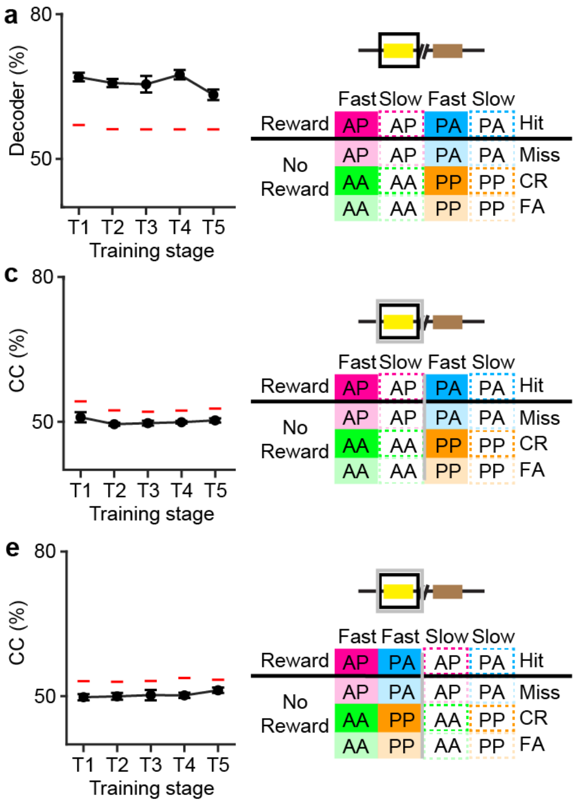

Supplementary Fig. 10. Population coding of reward prediction during sample and test periods and its relationship to stimulus coding. a, Linear decoder performance of sample period activity to rewarded conditions across training. **b,** Linear decoder performance of test period activity to rewarded conditions across training. **c,** Cross-condition performance of sample period activity trained to rewarded conditions and tested on stimulus direction conditions across training. **d,** Cross-condition performance of test period activity trained to rewarded conditions and tested on stimulus direction conditions across training. **e,** Cross-condition performance of sample period activity trained to rewarded conditions and tested on stimulus speed conditions across training. **f,** Cross-condition performance of test period activity trained to rewarded conditions and tested on stimulus speed conditions across training. Error bars = SEM. Red line = 95th percentile performance of the shuffled distribution.

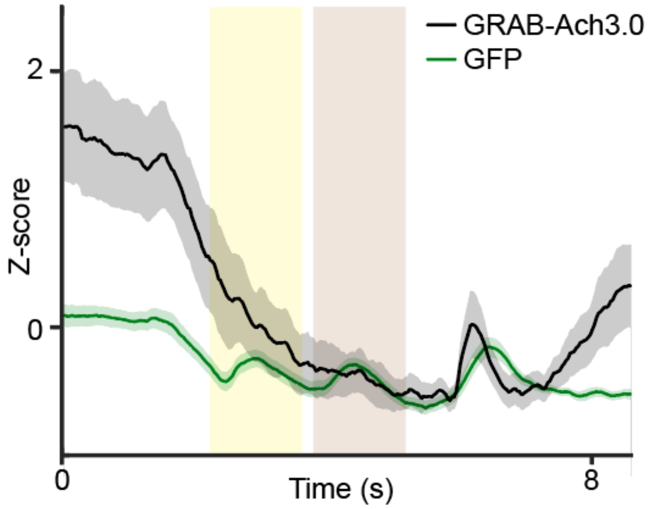

Supplementary Fig. 11. Validation of GRAB-Ach3.0. *Z*-scored fluorescence traces across the trial period during T2 sessions in task trained animals expressing either GRAB-Ach3.0 or GFP. *n* = 16 T2 sessions from 4 GRAB-Ach3.0 animals, 17 T2 sessions from 2 GFP animals. Shaded region = SEM.

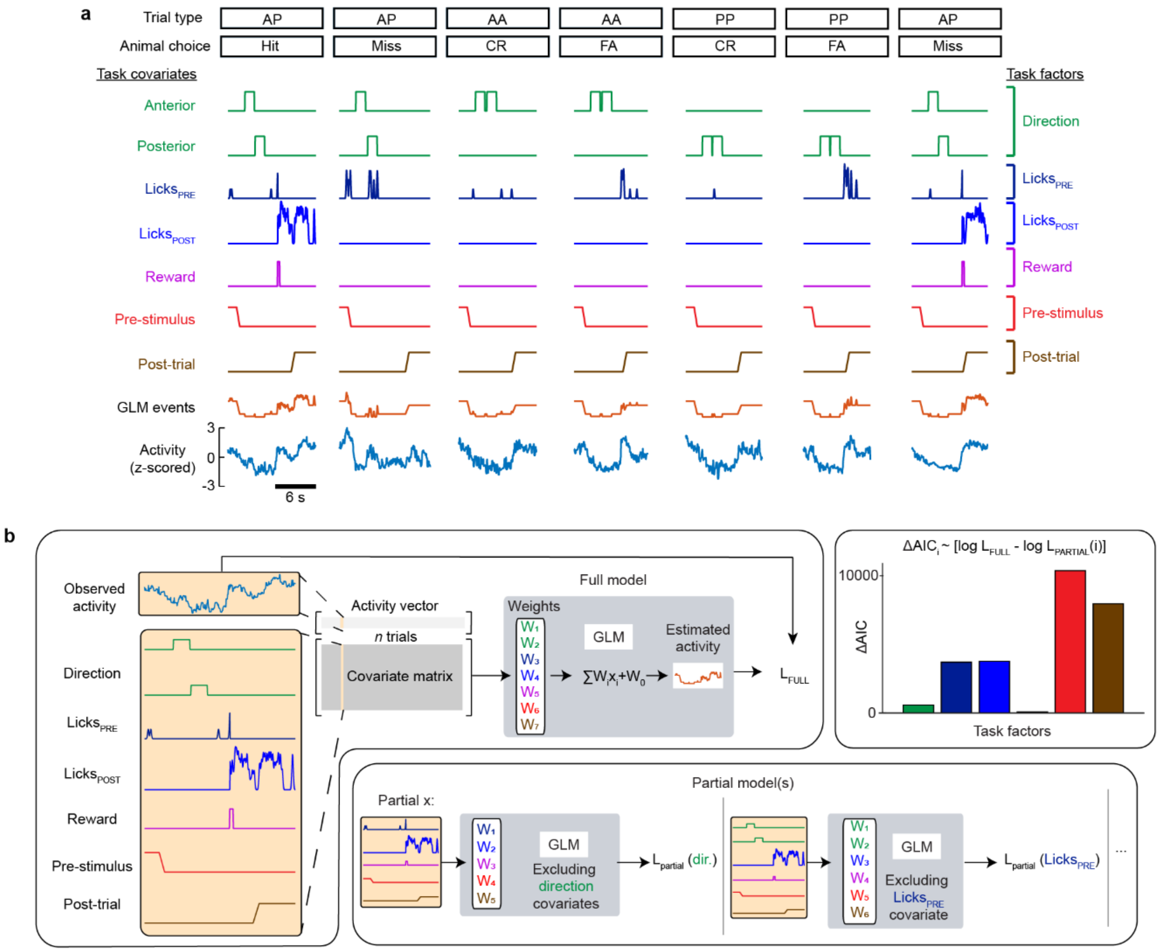

Supplementary Fig. 12. Task encoding of acetylcholine signals. a, Overview of covariate representations and their corresponding task factors used in the GLM for acetylcholine signals over six trials. **b,** Schematic of full and partial models used to calculate ΔAIC for individual task factors.

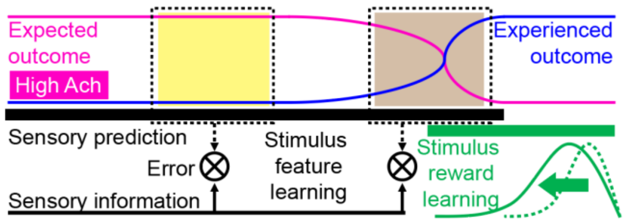

Supplementary Fig. 13. Conceptual model of predictive map in Prh. Prh forms a model of task-relevant stimulus information through error learning. Differences in predicted stimulus features elicit sensory prediction errors. Stimuls-reward associations emerge in a retrograde manner from reward outcomes and generalize to similar stimulus-reward contingencies. Expected outcomes are linked to experienced outcomes via a network space that is regulated by cholinergic signaling.

SUPPLEMENTARY NOTES

S1. Home-cage task training

In this study, two variations of the delayed non-match-to sample task were used to study the role of Prh in abstract sensory learning. To assay the effects of inactivating Prh on behavior, the home cage version of the task was designed to train animals in an unbiased manner. Task training occurred in a training module consisting of a narrow passageway that restricted movement of freely moving animals so that head position was consistent throughout training for reliable delivery of whisker stimulus and water reward (**Supplementary Fig. 2a**). For whisker stimulus, commercial grade sandpaper (3M; P100) was mounted along the outside edge of a 6 cm diameter rotor, attached to a stepper motor (Zaber) to deflect the whiskers. This was mounted onto a linear stage (Zaber) to place the rotor within whisker reach.

For lick sensing and water delivery, an angled dispensing needle (75165A22; McMaster-Carr) served as a water port. This was attached to a capacitive touch sensor (AT42QT1010; SparkFun) that dispensed 5-7 µL of water through a miniature solenoid valve (LHDA0531115H; The Lee Company). Unlike head-fixed behavior (**Supplementary Note S2**), persistent and impulsive licking was prevalent during freely moving behavior. Attempts to train home-cage animals to learn a go/no go stimulus-reward contingency were not successful due to impulsive licking (data not shown). For these reasons, a two-alternative forced choice (2AFC) task structure using two lick ports was employed for home-cage behavior. To further discourage impulsive licking, lick spouts were mounted onto a linear actuator (L12-P; Actuonix) and only presented to the animals during the report period. This differed from head-fixed training in which lick spouts were fixed always in reach of the animal. Air puffs were controlled using a 12V solenoid (EV-2-12; Clippard). Task training was performed using a custom written LabVIEW software (National Instruments) to control hardware and a data acquisition interface (USB-6008; National Instruments) for measuring licks, water delivery, and air puff delivery.

The task was designed for live-in conditions in which trials were self-initiated and task parameters automatically adjusted based on performance. A single training module was connected to three cages, each containing a singly-housed mouse. Mice were singly-housed to avoid social interactions that would interfere with equal access to task training. Head-fixed mice were similarly singly-housed to minimize potential damage to their implants. Cages were connected via passageways to a common meeting chamber. For each passageway, access to the training module via the meeting chamber was regulated by mechanical doors. These doors were controlled by servos operated by an Arduino microcontroller. Door closing was trigged by an infrared beam break sensor placed between the door and home cage in order to ensure that the door did not close while the animal was in the training module.

Access to home-cage training was scheduled similarly to head-fixed task conditions to ensure equivalent water deprivation periods, motivation levels, session duration, and trial numbers. Each animal gained daily access to the training module for two, two-hour sessions (**Supplementary Fig. 2b**). To ensure that each animal performed the task across all dark portions of light-dark cycle, the scheduled animal order was rotated daily. At the end of each session, the training module break beam sensor was deactivated to prevent trial initiation. A continuous train of air puffs was delivered into the chamber signaling the animal to exit and for the door to close behind them (**Supplementary Fig. 2c**). A USB radio frequency identification (RFID) reader above the meeting chamber was used to ensure that the correct animal accessed the training module at the properly scheduled time.

We tested whether Prh was required to learn the classification task presented in the system using chronic chemogenetic inactivation[2]. Advancement to successive training stages was contingent on pre-defined performance metrics that were applied uniformly to each animal (**Table 1**, **Supplementary Note S3**). Reporting of non-match vs. match conditions was carried out by two-alternative forced choice licking of water ports and reinforced by delivery of water reward. We stereotaxically localized whisker-related regions of Prh by first using anatomical tracing to identify sites that exhibit reciprocal connectivity between secondary somatosensory cortex (S2) (**Supplementary Fig. 1**). Experimental hM4Di+ animals (n = 9) received bilateral injections of AAV/9-*hSyn-dio-hM4Di-mCherry* and rAAV-*hSyn-Cre* into the targeted area (**Supplementary Fig. 3a**). Control hM4Di- animals (n = 13) either received no injection or bilateral sham injections of AAV/9-*hSyn-dio-mCherry* and rAAV-*hSyn-Cre.* All animals were placed in the home cage training system for up to six weeks (~80 training sessions) with Compound 21 provided in the drinking water to silence neurons in Prh[3]. Histology was performed at the end of behavior experiments to verify viral targeting of Prh. For some animals, the density of hM4Di-mCherry expression (74.9±3.0% of neurons, n = 4 animals) along with mRNA *Fos* expression were quantified to verify Prh silencing (**Supplementary Fig. 3b-c**). Individual hM4Di+ or hM4Di- animals showed a range of learning rates throughout the training period (**Supplementary Fig. 4**). However, the majority of hM4Di+ animals failed to demonstrate consistent learned behavior to advance past T2 (**Supplementary Fig. 3d**). hM4Di+ animals spent more trials in T1-T2 than hM4Di- animals (**Supplementary Fig. 3e**, 14,976±1,473 trials hM4Di+ animals vs. 11,058±1,512 trials hM4Di- animals; $P<0.05$, Student's t-test). This demonstrates that Prh is involved in sensory-guided task learning.

While home-cage training with animals under freely moving conditions enables high-throughput, unbiased assays of complex task learning, a limitation of this approach with respect to this study is that the behavioral conditions are not identical to the head-fixed conditions used for characterizing Prh calcium and Ach responses. Stimulus-action-outcomes relationships differ between the two task conditions. While the training module restricts the animals' movements, they are still free to employ a large number of motor strategies in the freely moving condition. The role of Prh in sensory learning has been demonstrated under multiple task conditions[4,5]. Our findings in the freely moving condition confirm this earlier evidence, reinforcing the idea that Prh supports sensory learning across multiple behaviors.

S2. Head-fixed task training

The head-fixed version of the task was designed to reliably image neuronal activity during learning under highly consistent, well-controlled stimulus conditions. A go/no go stimulus-reward contingency was employed to characterize activity patterns related to stimulus information with and without reward associations. Similar to home-cage training, the task was performed using a custom written LabVIEW software (National Instruments) to control hardware and a data acquisition interface (USB-6008; National Instruments) for measuring licks, water delivery, and air puff delivery. A water port was attached to a capacitive lick sensor (AT42QT1010; SparkFun) that dispenses 5 to 6 µL of water through a miniature solenoid valve (0127; Buekert). For the rotation stimulus, commercial grade sandpaper (3M; roughness: P100) was mounted along the outside edge of a 6 cm diameter rotor, attached to a stepper motor (Zaber) to deflect the whiskers which was mounted onto a linear stage (Zaber) to place the rotor within whisker reach.

Given the time demands of the experiment for operating the two-photon microscope through learning (~70 sessions, 2 sessions per day, 7 days per week), ensuring successful training

was a priority for animals undergoing imaging. Compared to the home-cage training, similar performance criteria were applied to advance animals through each stage of training (**Table 2**, **Supplementary Note S3**). However, given the natural variability in learning across individual animals, some training parameters were manually tuned to reinforce correct choice behavior and maximize training success (**Table 3, Supplementary Note S3-S4**).

S3. Training stages

The task settings defining each training stage in the home cage (**Table 1**) and head-fixed (**Table 2**) training task were largely similar with the following exceptions. For the head-fixed task, the proportion of non-match versus match trials were gradually changed from 0.9/0.1 to 0.5/0.5 (non-match/match) over the course of the first 5 T1 sessions. The purpose of this was to acclimate the animals to licking for reward and to avoid miss trials by providing a high proportion of rewarded (non-match) stimulus trials and gradually exposing animals to the non-rewarded (match) stimulus trials. For the home cage task, the proportion of non-match versus match trials were set to 0.5/0.5. During early T1 sessions, the maximum consecutive trials belonging to either match or non-match stimulus was set to 1. This meant that water reward alternated between each lick port in order to acclimate the animal to licking to each port. The target spout alternated between trials through four sub-stages which taught animals how to receive rewards and gradually introduced the moving parts of the task. In the first sub-stage, the texture was positioned against the training module but did not provide directional stimuli. Instead, animals were able to trigger a trial and lick when an audible tone was played in order to receive a water reward. With consistent lick responses, the delay between triggering a trial and the tone indicating the report period was increased from 100ms to 6s, approximating the time course of a trial with two stimuli and a 2s delay. In the second sub-stage, the sample and test stimuli were presented and the report period was still indicated with a tone. This tone was removed during the third sub-stage. The fourth sub-stage introduced linear movement of the texture, withdrawing it at the end of a trial and moving it to presentation position for the sample and test periods. The maximum number of consecutive trials with the same target spout was then increased to 3 in the fifth sub-stage to randomize the stimulus conditions.

During T4, the delay between the sample and test stimulus was gradually increased through a progression of sub-stages. An initial delay was used at the beginning of the session. Behavioral performance was measured every 15 trials. The delay was increased by a defined increment if performance exceeded >85% correct ($d' > 2.07$) over the past 15 trials up to a maximum of 2 seconds. If the overall performance for the session was $d'>1.68$, the animal advanced to the next T4 sub-stage in which the starting delay and increment was greater than that used in previous session. The rotor was withdrawn once animals could begin sessions with delays of 2 seconds. In general, head-fixed animals could readily adapt to the rotor withdrawal during the delay period. Initial piloting of the same training progression during home-cage task training suggested that animals had difficulty with adapting to this transition. For this reason, the training protocol in the home-cage task was modified to include a gradual withdrawal of the rotor occurring concurrently with the gradual increase in delay period.

During T5, delays were randomly varied between 2, 3, and 4 seconds for head-fixed animals to examine sequential activity across varying delay periods. In home-cage animals, the delay was fixed at 2 seconds. Finally, slow speed stimulus conditions were included for head-fixed task in order to measure activity related to relevant and irrelevant stimulus features but were not included during the home-cage task since the motivation of the latter was to broadly assay the dependence of Prh on task learning.

S4. Reinforcing correct choice

Due to the complexity of task conditions and stimuli, we observed that individual animals adopted a range of incorrect choice strategies early during task training. Occasionally, behavioral lapses were also observed in which animals demonstrated correct choice strategies across extended trial periods but then reverted to incorrect choice strategies. Incorrect choice strategies were categorized as report bias, primacy bias, and recency bias. A set of task parameters were included in the training protocol to identify and correct for these biases without changing the stimulus-reward contingency (**Table 3**).

For go/no-go behavior under head-fixed conditions, a report bias was defined as persistent licking of the lick port regardless of stimulus condition. For 2AFC version of the task used in the home cage training system, persistent licking of one of two lick ports regardless of stimulus condition was considered a report bias. Report bias primarily contributed to poor task performance early in training during T1 and was also occasionally observed at the beginning of behavior sessions in trained mice. For the go/no go head-fixed task, report biases were defined by a high fraction of total hit and false alarm trials. For 2AFC home cage task, report biases were defined a high fraction of hit and false alarm trials attributed to one of the two lick ports. Depending on the severity of the report bias, two corrective strategies were adopted. The first strategy is the use of punishment to discourage licking of the incorrect stimulus condition. Punishment consisted of a combination of time out and air puffs to the face. Initially introduced punishment was mild and gradually became more severe with longer time outs and multiple air puffs considered as more severe punishment. Tolerance for punishment can vary for individual animals (data not shown). For both task conditions, animals disengaged from the task if punishment was too aversive, resulting in miss trials. Punishment levels are reduced if misses increase. In addition to adjusting punishment levels, the probability of stimulus conditions was also adjusted to increase the frequency of the incorrect stimulus condition in order for animals to "practice" the correct response. Typically, non-match and match stimulus conditions were presented at 50% probability. This was increased up to 80% for the incorrect stimulus condition depending on the severity of the report bias.

A primacy stimulus bias represented incorrect choice strategies in which the animal responded based on whether the sample stimulus was A or P. In contrast, a recency stimulus bias represented incorrect choice strategies in which the animal responded based on whether the test stimulus was A or P. These biases were operationally defined as differences in performance between the two stimulus conditions belonging to the same category (AP vs. PA for non-match, AA vs. PP for match). Typically for each stimulus category, one of the two possible stimulus conditions is presented with 50% probability with respect to the other. To correct for primary or recency bias, the probability of stimulus conditions belonging to the same category was adjusted to increase the frequency of the incorrect stimulus condition in order for animals to "practice" the correct response.

SUPPLEMENTARY REFERENCES

1. Condylis, C. *et al.* Context-Dependent Sensory Processing across Primary and Secondary Somatosensory Cortex. *Neuron* **106**, 515-525.e5, doi:10.1016/j.neuron.2020.02.004 (2020).

2. Armbruster, B. N., Li, X., Pausch, M. H., Herlitze, S. & Roth, B. L. Evolving the lock to fit the key to create a family of G protein-coupled receptors potently activated by an inert ligand. *Proc. Natl. Acad. Sci. U. S. A.* **104**, 5163–5168, doi:10.1073/pnas.0700293104 (2007).

3. Chen, X. *et al.* The first structure-activity relationship studies for designer receptors exclusively activated by designer drugs. *ACS Chem. Neurosci.* **6**, 476–484, doi:10.1021/cn500325v (2015).

4. Suzuki, W. A. & Naya, Y. The perirhinal cortex. *Annu. Rev. Neurosci.* **37**, 39–53, doi:10.1146/annurev-neuro-071013-014207 (2014).

5. Miyashita, Y. Perirhinal circuits for memory processing. *Nat. Rev. Neurosci.* **20**, 577–592, doi:10.1038/s41583-019-0213-6 (2019).
