# Supplementary material for: Perirhinal cortex learns a predictive map of the task environment
Source: Nat Commun. 2024 Jul 2;15:5544. doi: 10.1038/s41467-024-47365-7 (PMC11219840; doi:10.1038/s41467-024-47365-7)
Supplement: Supplementary file 3 — Reporting Summary [file 41467_2024_47365_MOESM3_ESM.pdf]

nature portfolio

Corresponding author(s): Jerry Chen

Last updated by author(s): 1/29/2024

Reporting Summary

Nature Portfolio wishes to improve the reproducibility of the work that we publish. This form provides structure for consistency and transparency in reporting. For further information on Nature Portfolio policies, see our Editorial Policies and the Editorial Policy Checklist.

Statistics

For all statistical analyses, confirm that the following items are present in the figure legend, table legend, main text, or Methods section.

n/a	Confirmed	
☐	☒	The exact sample size (*n*) for each experimental group/condition, given as a discrete number and unit of measurement
☐	☒	A statement on whether measurements were taken from distinct samples or whether the same sample was measured repeatedly
☐	☒	The statistical test(s) used AND whether they are one- or two-sided<br>*Only common tests should be described solely by name; describe more complex techniques in the Methods section.*
☐	☒	A description of all covariates tested
☐	☒	A description of any assumptions or corrections, such as tests of normality and adjustment for multiple comparisons
☐	☒	A full description of the statistical parameters including central tendency (e.g. means) or other basic estimates (e.g. regression coefficient) AND variation (e.g. standard deviation) or associated estimates of uncertainty (e.g. confidence intervals)
☐	☒	For null hypothesis testing, the test statistic (e.g. *F*, *t*, *r*) with confidence intervals, effect sizes, degrees of freedom and *P* value noted<br>*Give P values as exact values whenever suitable.*
☒	☐	For Bayesian analysis, information on the choice of priors and Markov chain Monte Carlo settings
☒	☐	For hierarchical and complex designs, identification of the appropriate level for tests and full reporting of outcomes
☐	☒	Estimates of effect sizes (e.g. Cohen's *d*, Pearson's *r*), indicating how they were calculated

Our web collection on statistics for biologists contains articles on many of the points above.

Software and code

Policy information about availability of computer code

Data collection	Two-photon microscope data collection was performed using a customized LabVIEW-based FPGA program and custom C++-based software, Scope, which is available at http://rkscope.sourceforge.net. Custom LabVIEW (2019 SP1) programs were also used to conduct experiments and collect behavioral data.
Data analysis	MATLAB r2021b was used for processing two-photon data and home cage data. Fiji was used for registering two-photon data from different sessions. Autoencoder modeling was performed in Python and PyTorch. Code is available at github.com/ramonnogueira/AutoPerirhinal [https://zenodo.org/doi/10.5281/zenodo.10581525].

For manuscripts utilizing custom algorithms or software that are central to the research but not yet described in published literature, software must be made available to editors and reviewers. We strongly encourage code deposition in a community repository (e.g. GitHub). See the Nature Portfolio guidelines for submitting code & software for further information.

Data

Policy information about availability of data

All manuscripts must include a data availability statement. This statement should provide the following information, where applicable:

- Accession codes, unique identifiers, or web links for publicly available datasets
- A description of any restrictions on data availability
- For clinical datasets or third party data, please ensure that the statement adheres to our policy

Derived data generated in this study are available in a G-Node GIN repository [https://doi.org/10.12751/g-node.m36s4g]. Raw data are available from the corresponding authors upon reasonable request due to prohibitively large file sizes.

Research involving human participants, their data, or biological material

Policy information about studies with human participants or human data. See also policy information about sex, gender (identity/presentation), and sexual orientation and race, ethnicity and racism.

Reporting on sex and gender	N/A
Reporting on race, ethnicity, or other socially relevant groupings	N/A
Population characteristics	N/A
Recruitment	N/A
Ethics oversight	N/A

Note that full information on the approval of the study protocol must also be provided in the manuscript.

Field-specific reporting

Please select the one below that is the best fit for your research. If you are not sure, read the appropriate sections before making your selection.

☒ Life sciences ☐ Behavioural & social sciences ☐ Ecological, evolutionary & environmental sciences

For a reference copy of the document with all sections, see nature.com/documents/nr-reporting-summary-flat.pdf

Life sciences study design

All studies must disclose on these points even when the disclosure is negative.

Sample size	No statistical methods were used to predetermine sample size. The number of subjects used is comparable to previous studies (Chen et al., 2015 Nat. Neuroscience)
Data exclusions	Two-photon data from three animals were excluded. Image quality was too poor for one animal and two were unable to learn the task.
Replication	All head-fixed subjects were trained and imaged independently over consecutive sessions, the number of which was determined by the individual's performance, with a minimum of 26 sessions. Home cage subjects were trained independently across 3-5 systems for roughly 86 sessions per cohort.
Randomization	The stimulus conditions that were presented during each trial of the tasks were pseudo-randomly selected from four possibilities. For the head-fixed task, the proportion of non-match versus match trials was gradually changed from approximately 0.9/0.1 to 0.5/0.5 (non-match/match). For the home-cage task, the proportion of non-match versus match trials was set to 0.5/0.5 and adjusted for behavior shaping as described in the Methods.
Blinding	Blinding was used during data collection for home-cage task training involving hM4Di+ and control animals to ensure comparable treatment between animals. Data were unblinded during analysis to enable comparison between groups. Blinding was not performed for head-fixed task training where animals underwent the same treatment.

Reporting for specific materials, systems and methods

We require information from authors about some types of materials, experimental systems and methods used in many studies. Here, indicate whether each material, system or method listed is relevant to your study. If you are not sure if a list item applies to your research, read the appropriate section before selecting a response.

Materials & experimental systems

n/a	Involved in the study
☒	☐ Antibodies
☒	☐ Eukaryotic cell lines
☒	☐ Palaeontology and archaeology
☐	☒ Animals and other organisms
☒	☐ Clinical data
☒	☐ Dual use research of concern
☒	☐ Plants

Methods

n/a	Involved in the study
☒	☐ ChIP-seq
☒	☐ Flow cytometry
☒	☐ MRI-based neuroimaging

Animals and other research organisms

Policy information about studies involving animals; ARRIVE guidelines recommended for reporting animal research, and Sex and Gender in Research

Laboratory animals	C57Bl/6 mice aged 6-8 weeks at the time of surgery were used. Animals were singly-housed after window surgery to prevent damage to implants, and animals trained in the home cage system were singly-housed throughout the experiment. All animals were housed in a 12-hour reverse light cycle room with temperature 68-79 deg. F and humidity 30-70%.
Wild animals	The study did not involve wild animals.
Reporting on sex	Head-fixed behavior experiments in this study used all male mice. Home cage behavior experiments used female (n=6) and male (n=16) mice.
Field-collected samples	The study did not involve field-collected samples.
Ethics oversight	All experimental procedures were approved by the Institutional Animal Care and Use Committee for the Charles River Campus at Boston University.

Note that full information on the approval of the study protocol must also be provided in the manuscript.
